# Systemically Administered, Target Organ-Specific Therapies for Regenerative Medicine

**DOI:** 10.3390/ijms161023556

**Published:** 2015-09-30

**Authors:** Tero A. H. Järvinen, Ulrike May, Stuart Prince

**Affiliations:** 1 School of Medicine, University of Tampere, 33520 Tampere, Finland; E-Mails: ulrike.may@uta.fi (U.M.); stuart.prince@uta.fi (S.P.); 2 Department of Orthopedics & Traumatology, Tampere University Hospital, 33520 Tampere, Finland

**Keywords:** angiogenesis, tissue regeneration, *in vivo* phage display, decorin, vascular ZIP codes, regenerative medicine

## Abstract

Growth factors and other agents that could potentially enhance tissue regeneration have been identified, but their therapeutic value in clinical medicine has been limited for reasons such as difficulty to maintain bioactivity of locally applied therapeutics in the protease-rich environment of regenerating tissues. Although human diseases are treated with systemically administered drugs in general, all current efforts aimed at enhancing tissue repair with biological drugs have been based on their local application. The systemic administration of growth factors has been ruled out due to concerns about their safety. These concerns are warranted. In addition, only a small proportion of systemically administered drugs reach their intended target. Selective delivery of the drug to the target tissue and use of functional protein domains capable of penetrating cells and tissues could alleviate these problems in certain circumstances. We will present in this review a novel approach utilizing unique molecular fingerprints (“Zip/postal codes”) in the vasculature of regenerating tissues that allows target organ-specific delivery of systemically administered therapeutic molecules by affinity-based physical targeting (using peptides or antibodies as an “address tag”) to injured tissues undergoing repair. The desired outcome of targeted therapies is increased local accumulation and lower systemic concentration of the therapeutic payload. We believe that the physical targeting of systemically administered therapeutic molecules could be rapidly adapted in the field of regenerative medicine.

## 1. Local *vs.* Systemic Drug Delivery in Regenerative Medicine

Adult tissues respond to injury differently. Some tissues, such as the bone, repair injuries with tissue that is identical to the original tissue. However, most tissues respond by undergoing a repair process that only partially restores the original tissue with the rest replaced by non-functioning, fibrotic scar tissue [1,2].

Numerous growth factors and other agents that could potentially enhance tissue regeneration have been identified, but their therapeutic application has been rather limited in clinical medicine [1,3,4]. There are several reasons for their limited use: it is difficult to maintain bioactivity of locally applied therapeutic agents in regenerating tissue because of lack of retention of the agent, poor tissue penetration, and instability of protein therapeutics in the protease-rich environment of the injured tissue [4,5]. Moreover, most injuries are not accessible with topical application of therapeutic molecules and multiple sites (tissues) of injury further limit the usefulness of local treatment.

Strikingly, all current efforts aimed at enhancing tissue repair with biologic drugs have been based on local application of therapeutic molecules to the injured site [5,6]. Although human diseases are treated with systemically administered drugs in general, systemic administration of growth factors has been ruled out due to concerns about their systemic use and potential safety. These concerns are warranted because the major problems in systemic drug therapy are that only a small proportion of administered drug reaches its intended target site(s). In addition, large molecules such as antibodies are poor at penetrating tissues and do not always reach the actual target cells [7,8,9,10]. Selective delivery of the drug to the target tissue and use of functional protein domains, such as cell penetrating peptides, capable of penetrating cells and tissues could alleviate some of these problems [10,11,12,13].

## 2. Vascular Heterogeneity—“Zip Codes” in Vasculature

**Figure 1:**
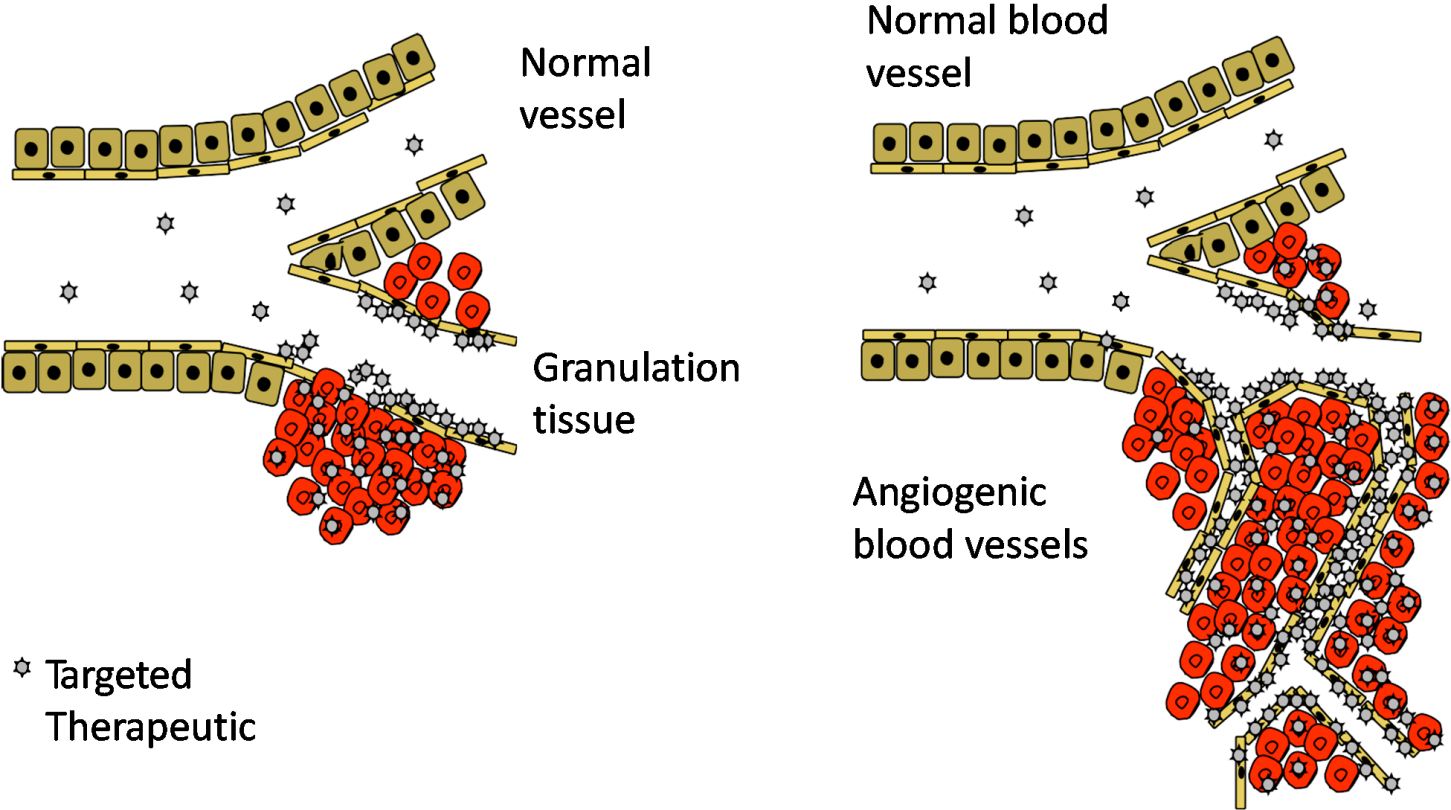
Target-specific drug therapies in tissue regeneration. Molecular fingerprints (“Zip/postal codes”) in the angiogenic vasculature of the regenerating tissues allow target organ-specific delivery of the systemically administered therapeutic molecules by affinity-based physical targeting (using peptides or antibodies as an “address tag”) to injured tissues undergoing repair. The desired outcome of the targeted therapies is similar to topical application: increased local accumulation and lower systemic concentration of the therapeutic payload.

Our increased understanding of the structure of blood vessels on the molecular level has revealed a practical possibility for organ-specific therapeutic treatment of various human diseases with systemically administered drugs [9,14]. Recent research has shown that each organ has unique molecular structures in its blood vessels (“vascular ZIP codes”) [9,14,15,16,17] (Figure 1). Each organ confers endothelial cells (ECs) in it with their “organotypic”, *i.e.*, unique tissue-specific, features [9,14,15,18]. Clusters of transcription factors, angiocrine growth factors, adhesion molecules and chemokines are expressed in unique combinations by the ECs of each organ and the blood vessels in each organ can be defined [15]. Furthermore, various diseases display disease-specific signatures on the vasculature of the diseased tissue [19]. This is especially evident for diseases such as different cancers, or tissue injury, which induce new blood vessels by angiogenesis [11]. The angiogenic blood vessels, in turn, are structurally distinct from the pre-existing blood vessels in the body [11,20]; a fact also highlighted by different types of stem cells being recruited to take part in the process [21].

The disease and organ-specific molecular “Zip codes” in blood vessels can be utilized for target organ-specific delivery of therapeutic molecules by affinity ligands [9,11,14,19]. Affinity-based physical targeting (synaphic, pathotrophic, or active targeting) makes use of these vascular ZIP codes, *i.e.*, molecular markers that are specifically expressed at the target, and not elsewhere in the body, to accomplish selective targeting of systemically administered drugs to the target organ [11]. The desired outcome of the synaphic targeting is increased local accumulation and lower systemic concentration of the therapeutic payload [11].

## 3. *In Vivo* Phage Display

**Figure 2:**
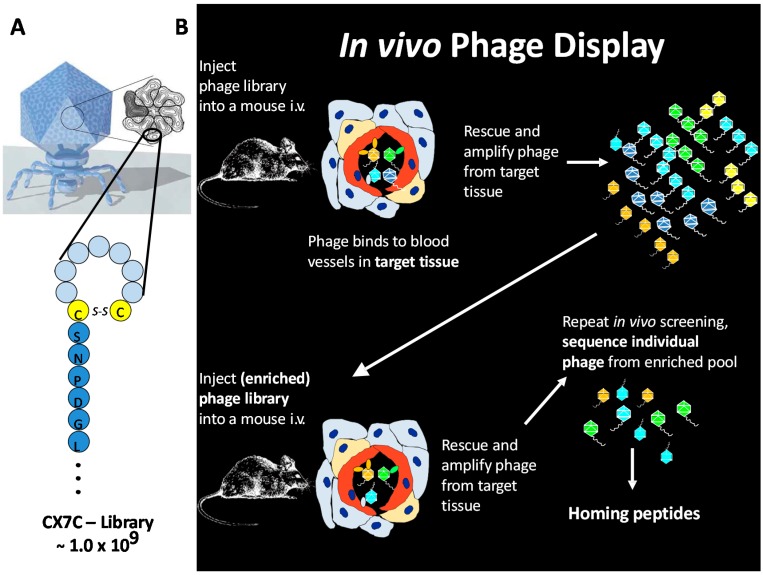
Schematic presentation of the principle of *in vivo* phage display. (**A**) A cyclic CX7C-peptide library is cloned onto the C-terminus of phage coat protein and 415 copies expressed per T7 phage via Select 415-1b; and (**B**) the phage library is injected into the circulation. As the homing peptides on the phage bind to endothelium in the tissues, there is an enrichment of phage that binds to the endothelium of the target tissue. Target tissue is homogenized, cell suspensions prepared, and the bound phage rescued and amplified by adding *E. coli*. The amplified phage pool recovered from the target tissue is re-injected into mice at a similar disease stage, and the screening cycle repeated several times to ensure phage clones that specifically bind (*i.e.*, home) towards target are recovered. A set of phage clones is collected from the homing phage population that shows enrichment towards the target tissue. The peptide-encoding DNA inserts are amplified by PCR, and the PCR products sequenced. Modified from Ruoslahti [19].

Vascular “ZIP codes” can be easily probed by *in vivo* phage display, a method first reported by Erkki Ruoslahti’s group in 1996 [14]. *In vivo* phage display allows unbiased exploration of vascular diversity by random peptide libraries expressed in bacteriophage [22] (Figure 2). Phage display is a powerful method for peptide library screening that provides a physical linkage between peptides (*i.e.*, the phenotype), which are displayed on the surface of a bacteriophage particle, and the encoding DNA (genotype) [22,23].

Bacteriophage can be genetically modified to incorporate random protein sequences as fusions with the coat proteins at a diversity of billions of variants per library, close to the total number of possible permutations of a random amino acid sequence [22]. The outcome of generating a random phage library is a pool of billions of bacteriophages all identical to each other except for the protein motif expressed at the end of its coat protein. For *in vivo* selection, a library of phage displaying random peptides is injected systemically into the animals, followed by removal of target organ, amplification of the bound phage from the target organ, and subjecting the amplified pool to another round of selection in new animals [22]. *In vivo* peptide phage screening combines subtractive elements (removal of phage displaying pan-specific peptides by all other organs of the body except the given target organ) with positive selection at the target tissue [22]. *In vivo* phage display offers a unique opportunity to screen for potentially billions of protein-based drug candidates simultaneously in an *in vivo* setting [22]. Combination of *in vivo* phage display and bacterial 2-hybridization allows simultaneous identification of ligand-receptor pairs for target organ-specific targeting [24].

So far *in vivo* phage display has been used for several purposes in the field of regenerative medicine: to identify peptides capable of homing to the angiogenic blood vessels forming at injured tissues [20] and for the identification and illumination of neural structures during surgery [25].

## 4. Angiogenesis—An Opportunity for Vascular Targeting in Regenerative Medicine

Angiogenesis is a vital requirement for wound healing because the formation of new blood vessels allows a variety of mediators, nutrients, and oxygen to reach the healing tissue [1,2,3,26]. Angiogenesis is driven by tissue hypoxia, and inflammation, as both surviving cells and the inflammatory cells that have invaded the injured area secrete a large number of angiogenic growth factors [1,3,26,27]. During regeneration of injured tissue, angiogenic capillary sprouts invade the fibrin/fibronectin-rich clot and within a few days organize into a thick microvascular network throughout the granulation tissue [1,2,3,26,28]. The name, granulation tissue, actually derives from the granular appearance of abundant, newly formed blood vessels. As these newly formed blood vessels essentially fill up the granulation tissue forming at the site of injury and they differ in molecular structure from pre-existing vasculature, they provide an opportunity for vascular targeting of systemically administered drugs for purposes of enhancing the tissue regeneration process [20,29,30].

## 5. CRK and CAR Vascular Homing Peptides for Regenerative Medicine

We have reasoned that it could be possible to deliver systemically administered drugs into regenerating tissues in a target organ-specific fashion independent of the location of the injury in the body [20]. For that goal, we have probed injured tissues during angiogenesis with *in vivo* phage display to identify protein motifs capable of homing to the injured tissue after systemic administration [20]. We have identified two peptides that selectively target injured tissues: CARSKNKDC (CAR) and CRKDKC (CRK) [20]. CAR is homologous to heparin-binding sites in various proteins and shows the highest homology with the main heparin-binding site of the known angiogenic growth factor, bone morphogenetic protein 4 (BMP4). In line with that heparin binding site, CAR peptide binds to cell surface heparan sulfate proteoglycans (HSPGs) and utilizes HSPGs for efficient cell and tissue penetration [20], whereas the CRK peptide is not capable of cell and tissue penetration [20,31]. CRK peptide, in turn, has structural similarity to segments in thrombospondin type 1 and 3 repeats identified in a large number of proteins. These two vascular homing peptides have a distinct difference in their targeting ability; CAR peptide shows a preference for early stages of tissue regeneration, whereas CRK favors injured tissues at later stages of healing [20].

## 6. Conjugated Delivery

**Figure 3:**
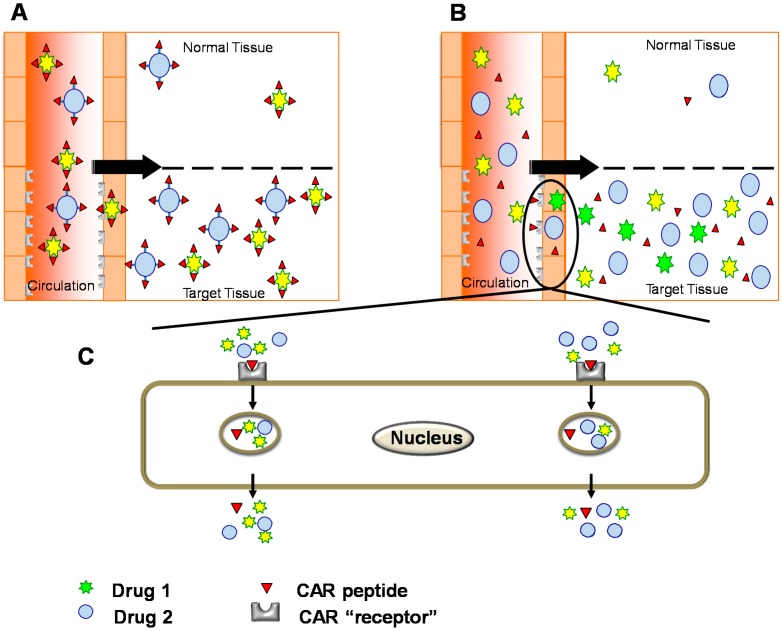
Systemically administered, targeted therapies: (**A**) The drugs are conjugated physically to the targeting device in conventional drug targeting. For protein-based therapeutics, targeting domain and therapeutic molecule are fused together as a recombinant protein with enhanced activity and tissue-specificity in conjugated delivery; (**B**,**C**) Bystander effect: Compounds co-injected with tissue-penetrating homing peptides are transported across the vessel wall and through tissue together with the peptides [12,42,43,44,45]. No physical conjugation is needed between the targeting peptide and drug; the cell penetrating homing peptide “sweeps” co-injected drugs to its target (homing) tissue in tissue-specific fashion [12,42,45].

Conventional tissue-specific delivery has called for conjugation of a targeting element to the therapeutic molecule to obtain a targeted therapeutic molecule (Figure 3). The conjugation can be either done by chemical conjugation of the two functional domains or by expressing them together as a recombinant fusion protein. Prime examples of such recombinant fusion proteins include interleukin-10 (IL-10) fused together with the antibody scFv F8 (F8-IL10, *i.e.*, Dekavil) that recognizes a domain of fibronectin expressed exclusively on inflammatory neovasculature [32,33,34,35]. This molecule is in clinical trials to treat arthritis [32,33,34,35]. Other examples include Angiopep, a peptide used to target nanoparticles loaded with therapeutic molecules to brains in e.g., Parkinson’s disease [13] and the tumor homing NRG-peptide fused together with tumor necrosis factor α (TNFα) to induce anti-tumor and toxic reactions towards tumor cells [36,37].

The half-life of the most common targeting element, peptides, is usually short in circulation and their binding affinity for their respective receptor relatively low. Both of these features can be substantially improved by fusing the peptides to become part of a larger recombinant protein or by coating cells or nanoparticles with multiple copies of the peptide [11,38]. For example, we have obtained preliminary evidence that incorporation of CAR peptide to therapeutic proteins enhances CAR peptide’s binding efficacy to its receptors to a nanomolar-level, and more so if the recombinant fusion protein is multimerized (expressed as a dimer). One-to-one peptide-payload conjugates can still be quite effective in targeting and avoiding side-effects in healthy tissues despite the large size difference between the peptide and the therapeutic domain, particularly when the peptide is used to augment an inherent affinity of the payload to the target [36,37,39]. A prime example of such complementary targeting is TNFα targeted to tumors with the NRG tumor-homing peptide; such a conjugate is now in phase 3 clinical trials [36,37], and CAR-peptide targeted decorin [39]. Decorin has been reported to home to angiogenic vasculature through a core protein-dependent interaction [40], but despite its inherent homing ability, the CAR peptide was able to enhance the accumulation of the fusion protein to sites of angiogenesis approximately 500% over native decorin [39]. An illustrative example of using multiple copies of short targeting peptides is by “painting” the intended therapeutic domain with multiple copies of the peptide. Such an approach is utilized when targeting stem cells to desired loci by “painting” the stem cells with multiple copies of the homing peptide [38,41]. Both CAR and CRK peptides have been used successfully to target mesenchymal stem cells to infarcted myocardium after “painting” the cells with multiple copies of the peptide [38,41]. The benefit of using homing peptides that are also cell penetrating peptides (CPPs) is that such a peptide greatly increases the transport of the drug to the extravascular tissue [10,20,39] (Figure 3).

Even more remarkable is the fact that the targeting domain can enhance the activity of the therapeutic molecule [39]. We have found that a vascular homing peptide for a tissue or lesion often also binds to the corresponding parenchymal cells [11], and this is the case with CAR, which also recognizes the granulation tissue in wounds [39]. This localization of the peptide receptor drives the translocation of the blood vessel-bound peptide into the injured tissue, where the therapeutic payload can exert its functions more potently as it is anchored on the target cells. We were able to show that the anchorage to target cells provided by CAR peptide’s fusion to decorin, enhanced decorin’s biological potency to neutralize transforming growth factor-β (TGF-β) [39]. Later, Hubbell *et al.* generated “super” growth factors, *i.e.*, more potent growth factors than native ones, simply by fusing them with the heparin-binding domain of BMP2 (analogous to the CAR peptide, which is a close homologue to the heparin binding domain of BMP4) [6].

One of the benefits of using short peptides as targeting elements, is that the peptides are unlikely to be immunogenic in themselves because they are simply too small. For example, they are a fraction of the size of the highly variable complementary determining regions (CDRs) of therapeutic antibodies, which generally have an excellent drug safety and low immune reaction record [10]. Furthermore, when analyzing the homology of homing peptides (by BLAST), these short and stable cyclic peptides typically show high levels of homology to parts of native proteins found across a range of species indicating that the functional domain in the homing peptide is conserved between species (as is the case for both CRK and CAR, for example) [20]. This would predict high tolerance of the peptide by the immune system.

These pharmacological properties highlight the importance of advancing multi-functional, systemically administered, target-seeking recombinant fusion proteins. However, unless the target of a homing peptide is known to be unimportant for function of the immune system, cell proliferation, angiogenesis, *etc.*, one cannot rule out the possibility of adverse effects caused by high doses of a homing peptide binding to its target receptor. Thus, it is necessary to elucidate the target and mechanism of homing peptides, and to investigate their safety.

## 7. Bystander Effect

**Figure 4:**
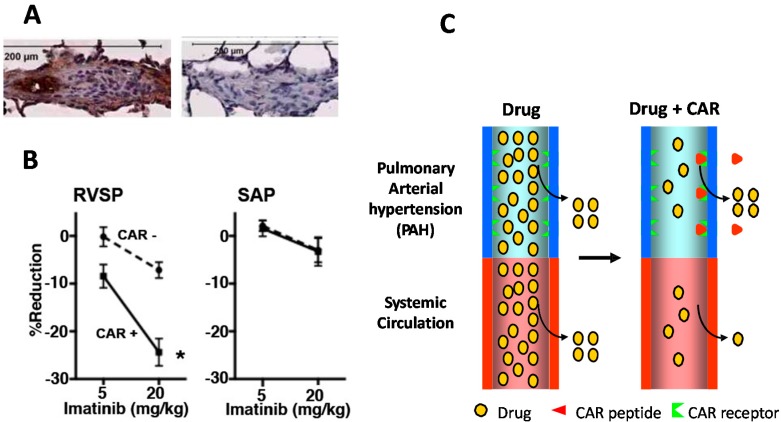
CAR peptide homes to inflammatory vasculature in pulmonary arterial hypertension (PAH) and induced target-tissue selective vasodilation in PAH. (**A**) PAH was induced in rats by a single subcutaneous injection of SU5416 and three week exposure to hypoxia (10% O_2_) followed by six weeks of normoxia [42,47]. CAR peptide accumulates in remodeled pulmonary arteries (A/C: occlusive neointimal formation) [42,47]. Little signal was detected in lung lesions of the PAH rats administered with CAR mutant peptide; (**B**) CAR peptide was given orally (p.o.) at the same time with Imatinib (Gleevec). CAR peptide makes drugs tissue-selective in PAH, *i.e.*, the drugs are substantially more potent in the pulmonary vasculature (RVSP) where the PAH takes place, whereas the blood pressure lowering effect induced by CAR co-administration is unchanged on the systemic side of the circulation (SAP), * *p* < 0.05; and (**C**) Schematic presentation of targeted delivery by the bystander effect induced by vascular homing peptide CAR.

The latest results have revealed a tissue-penetrating transport pathway for certain targeting peptides that can be used to enhance drug targeting without requiring any conjugation between the therapeutic and targeting domains [43,44,45] (Figure 3). The essential features of the system called the “bystander effect” were first elucidated using a new RGD (an integrin binding sequence) peptide, termed iRGD because it internalizes into target cells [43,45] (Figure 3 and Figure 4). Like conventional RGD peptides [46], iRGD accumulates at tumor vasculature as a result of integrin binding; but it is then cleaved by a protease to unmask a second binding motif, a so-called CendR motif (consensus: R/KXXR/K) [43,44,45]. The CendR motif binds to neuropilin-1, which activates a transport tissue-penetration and cell internalization pathway [43,44,45]. Remarkably, the CendR pathway is a bulk transport system. Once activated, it will sweep along any molecule or nanoparticle (“Bystander effect”) that is present in the environment (Figure 3). Thus, it is not necessary to couple iRGD to the compound to be targeted; the therapeutic molecule can be simply co-injected with the vascular homing peptide capable of inducing the bystander effect, which will sweep the molecule into the target organ [42,43,44,45] (Figure 3).

We have recently noted a bystander effect for a vascular homing and cell penetrating peptide that does not contain the CendR-motif [42] (Figure 3 and Figure 4). We have shown that the CAR peptide, that does not have a CendR-sequence in it [42], is a very potent inducer of a bystander effect in pulmonary hypertension (PAH) [42] (Figure 4). Namely, CAR peptide homes to the inflammatory vasculature in a rat model of PAH and penetrates to inflammatory lung parenchyme [42,47] (Figure 4). We have shown that it can convert any vasodilator drug to become a target organ-specific vasodilator in that model of PAH; when a low dose of vasodilator is given together with CAR peptide, the drug accumulates in the diseased lungs and lowers the hypertension selectively only in the pulmonary circulation and leaves the systemic circulation unaffected for target organ-specific vasodilation [42] (Figure 4). It remains to be determined whether CAR- or CendR-peptides induce a bystander effect that could be used for therapeutic advantage in regenerative medicine.

## 8. CAR-Decorin—Target Organ-Specific Systemically Administered Anti-Fibrotic Molecule

**Figure 5:**
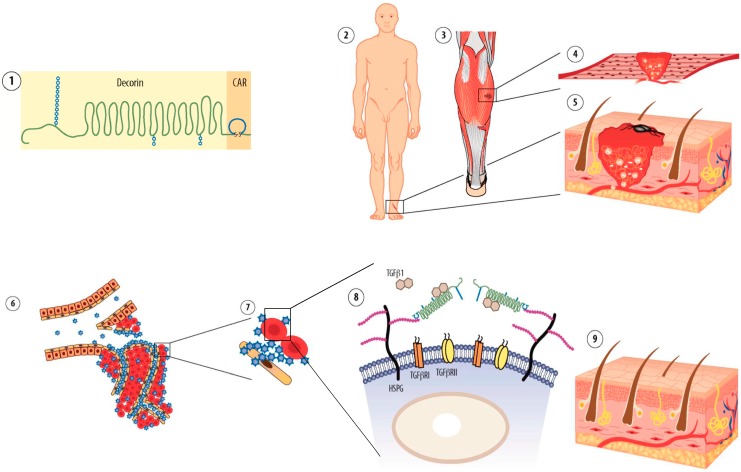
Schematic representation of the mechanism of action of the systemically administered, target organ-specific anti-fibrotic molecule, CAR-decorin. CAR-decorin (**1**) is a systemically administered, target-seeking, multi-functional biotherapeutic that inhibits scar formation. The molecule can be targeted to injury taking place at any organ of the body (**2**, **3**) (or multiple organs simultaneously). The CAR homing peptide targets angiogenic vasculature, which forms at the site of the injury (**4**, **5**). The peptide (and any payload attached to it; blue stars) then extravasates into surrounding tissue (**6**), where it binds to its receptor(s) on the cell surface of the scar producing fibroblasts (**7**). CAR binding to heparan sulfate proteoglycans (HSPGs) provides docking sites in the proximity of the main scar-inducing growth factors TGF-β1 and TGF-β2 (**8**) facilitating the neutralization of these growth factors by the therapeutic part of the molecule, decorin (**8**). This mechanism results in therapeutic response (**9**) seen as reduced scar formation in the skin [39]. Picture by Helena Schmidt; reproduced after modification with permission from [57], Copyright 2011 Finnish Medical Journal Duodecim.

**Figure 6:**
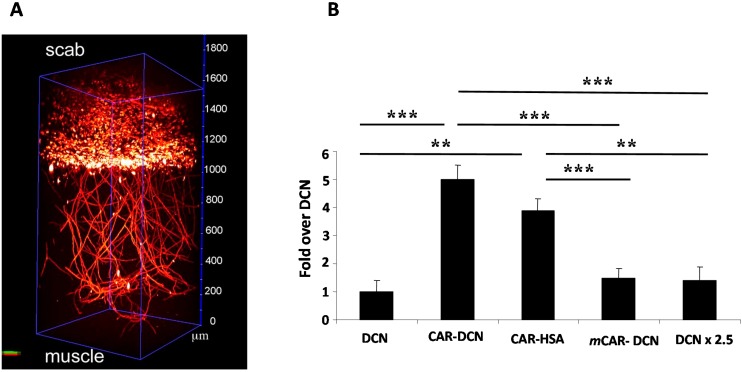
Targeted delivery of systemically administered therapeutics to the regenerating tissues. (**A**) 2-Photon fluorescence microscopy of vascular homing peptide accumulation to wound vasculature illustrates the specificity of the vascular targeting [30]. 2-Photon fluorescence microscope allows 3-D imaging of whole tissues [30]; and (**B**) Mice with full thickness skin wounds received an i.v. injection of His-tagged fusion proteins. The location of decorin was determined with an anti–His-tag antibody. The wounds of mice injected with non-targeted decorin (DCN) or mutant CAR peptide coupled decorin (*m*CAR-DCN) were weakly positive, whereas strong wound staining was observed in CAR-DCN injected mice [39]. The accumulation of CAR fused to human serum albumin (CAR-HSA) in the wounds confirmed the ability of the CAR peptide to enhance targeting to the wounds in fusion proteins other than DCN [39]. No staining was observed in normal skeletal muscle underlying the skin wounds of mice treated with any of the decorins. The staining was quantified by digital histology. The statistical significance was examined with ANOVA; ** *p* ≤ 0.01, *** *p* < 0.001 [39].

Decorin (DCN) is the best characterized member of the extracellular small leucine-rich proteoglycan family. It is present in a variety of connective tissues, typically in association with or “decorating” collagen fibrils, and has substantial interest to clinical medicine owing to its well established anti-fibrotic and pro-regenerative effects [48,49,50]. DCN is a natural inhibitor of TGF-β that shuts down the TGF-β responses related to injury, cancer growth and inflammation [39,49,50,51,52,53]. In addition to blocking TGF-β, DCN also inhibits other important inducers of fibrosis/scarring; myostatin and connective tissue growth factor (CTGF/CCN2) [29,50,54,55,56]. The ability of decorin to prevent scar formation and fibrosis and to simultaneously promote tissue regeneration has been demonstrated in a large number of tissue injuries and diseases [49,50,51]. We have recently developed a systemically administered, targeted, e.g., inflammation and angiogenesis homing version of DCN core protein [39] (Figure 5). The angiogenesis and inflammation specificity of our enhanced DCN core protein is obtained by a fusion to the CAR homing peptide that functions as an address tag in the fusion protein and delivers systemically administered DCN to angiogenic and inflammatory vasculature [29,39] (Figure 5 and Figure 6). CAR peptide can deliver increased amounts of DCN in a tissue-specific manner with a significant therapeutic advantage over ordinary DCN core protein in healing skin wounds [29,39] (Figure 5 and Figure 6). Furthermore, the fusion of CAR to recombinant DCN enhances its neutralization effect on TGF-β [39]. The molecular explanation for the enhanced biological activity of CAR-DCN is that the CAR peptide binds to HSPGs on the target cells. TGF-β1, and TGF-β2 in turn, also bind heparan sulfate and HSPG binding increases their biological activity [29,39] (Figure 5). Thus, CAR mediated binding of CAR–DCN to HSPGs may enhance the neutralizing effect of the fusion protein by bringing it into the proximity of the HSPG-binding TGF-βs [29,39] (Figure 5).

## 9. Future Perspectives

In diseases affecting lungs, the CAR peptide has also recently been shown to target inflammatory vasculature (Figure 4) [42,47,57,58,59,60,61] and to deliver different pharmaceutical agents in a target organ-specific fashion to diseased lungs (Figure 4) [42]. Furthermore, both CAR and CRK peptides have been used to target mesenchymal stem cells to infarcted myocardium [38]. Thus, CAR-targeted therapeutic molecules could also be useful in the treatment of chronic, inflammatory diseases (outside of healing wounds), or in diseases where there is angiogenesis or inflammation affecting the local vasculature.
